# Correction to ‘Characterization of an antagonistic switch between histone H3 lysine 27 methylation and acetylation in the transcriptional regulation of Polycomb group target genes’

**DOI:** 10.1093/nar/gkab625

**Published:** 2021-07-31

**Authors:** Diego Pasini, Martina Malatesta, Hye Ryung Jung, Julian Walfridsson, Anton Willer, Linda Olsson, Julie Skotte, Anton Wutz, Bo Porse, Ole Nørregaard Jensen, Kristian Helin

**Affiliations:** Biotech Research and Innovation Centre (BRIC), University of Copenhagen, Ole Maaløes Vej 5, 2200 Copenhagen, Denmark; Centre for Epigenetics, University of Copenhagen, Ole Maaløes Vej 5, 2200 Copenhagen, Denmark; Biotech Research and Innovation Centre (BRIC), University of Copenhagen, Ole Maaløes Vej 5, 2200 Copenhagen, Denmark; Centre for Epigenetics, University of Copenhagen, Ole Maaløes Vej 5, 2200 Copenhagen, Denmark; Centre for Epigenetics, University of Copenhagen, Ole Maaløes Vej 5, 2200 Copenhagen, Denmark; Department of Biochemistry and Molecular Biology, University of Southern Denmark, Campusvej 55, 5230 Odense, Denmark; Biotech Research and Innovation Centre (BRIC), University of Copenhagen, Ole Maaløes Vej 5, 2200 Copenhagen, Denmark; Centre for Epigenetics, University of Copenhagen, Ole Maaløes Vej 5, 2200 Copenhagen, Denmark; Biotech Research and Innovation Centre (BRIC), University of Copenhagen, Ole Maaløes Vej 5, 2200 Copenhagen, Denmark; Department of Clinical Biochemistry, Section for Gene Therapy Research, Copenhagen University Hospital, Blegdamsvej 9, 2100 Copenhagen, Denmark; Biotech Research and Innovation Centre (BRIC), University of Copenhagen, Ole Maaløes Vej 5, 2200 Copenhagen, Denmark; Centre for Epigenetics, University of Copenhagen, Ole Maaløes Vej 5, 2200 Copenhagen, Denmark; Biotech Research and Innovation Centre (BRIC), University of Copenhagen, Ole Maaløes Vej 5, 2200 Copenhagen, Denmark; Centre for Epigenetics, University of Copenhagen, Ole Maaløes Vej 5, 2200 Copenhagen, Denmark; Research Institute of Molecular Pathology, Dr. Bohr-Gasse 7, Vienna, Austria; Biotech Research and Innovation Centre (BRIC), University of Copenhagen, Ole Maaløes Vej 5, 2200 Copenhagen, Denmark; Department of Clinical Biochemistry, Section for Gene Therapy Research, Copenhagen University Hospital, Blegdamsvej 9, 2100 Copenhagen, Denmark; Centre for Epigenetics, University of Copenhagen, Ole Maaløes Vej 5, 2200 Copenhagen, Denmark; Department of Biochemistry and Molecular Biology, University of Southern Denmark, Campusvej 55, 5230 Odense, Denmark; Biotech Research and Innovation Centre (BRIC), University of Copenhagen, Ole Maaløes Vej 5, 2200 Copenhagen, Denmark; Centre for Epigenetics, University of Copenhagen, Ole Maaløes Vej 5, 2200 Copenhagen, Denmark

In Figure 5 of article (1), the authors have inadvertently duplicated the Vinculin blot in panel C.

**Figure 5.**
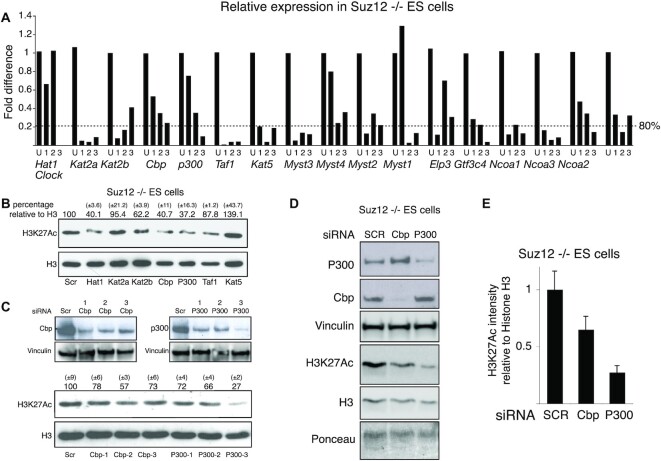
p300 and Cbp are required for efficient H3K27 acetylation in *Suz12* KO ES cells. (**A**) qPCR expression analyses of the indicated genes in *Suz12* KO ES cells transfected for 48 h with the indicated siRNA oligos. ‘U’ indicates the control siRNA oligo carrying a scrambled oligoribonucleotide sequence. (**B**) Western blot analyses of histones purified from *Suz12* KO ES cells transfected with the indicated siRNA oligos using the indicated antibodies. H3 is presented as loading control. Quantification of the H3/H3K27Ac signal is indicated above each lane. A scrambled siRNA oligo (SCR) was used as negative control. (**C** and **D**) Western blot analyses of protein extracts and of purified histones from *Suz12* KO ES cells transfected with the indicated siRNA oligos using the indicated antibodies. Vinculin, Ponceau staining and H3 are presented as loading controls. A scrambled siRNA oligo (SCR) was used as negative control. Quantification of the H3/H3K27Ac signal of western blots presented in ‘C’ is indicated above each lane. (**E**) Average quantification of the H3/H3K27Ac signals between the two independent siRNA experiments presented in C and D.

Below are the original raw image and a new Figure 5. In the raw image, the first four lanes (Lanes 1–4) belong to the left panel of original Figure 5C. The next four (Lanes 5–8) are the ones that need to be used to substitute the wrong duplicated vinculin panel (right top panel of original Figure 5C). The last two lanes (Lanes 9–10) were not be included in Figure 5.

This error does not affect the results, discussion and conclusions presented in the article.


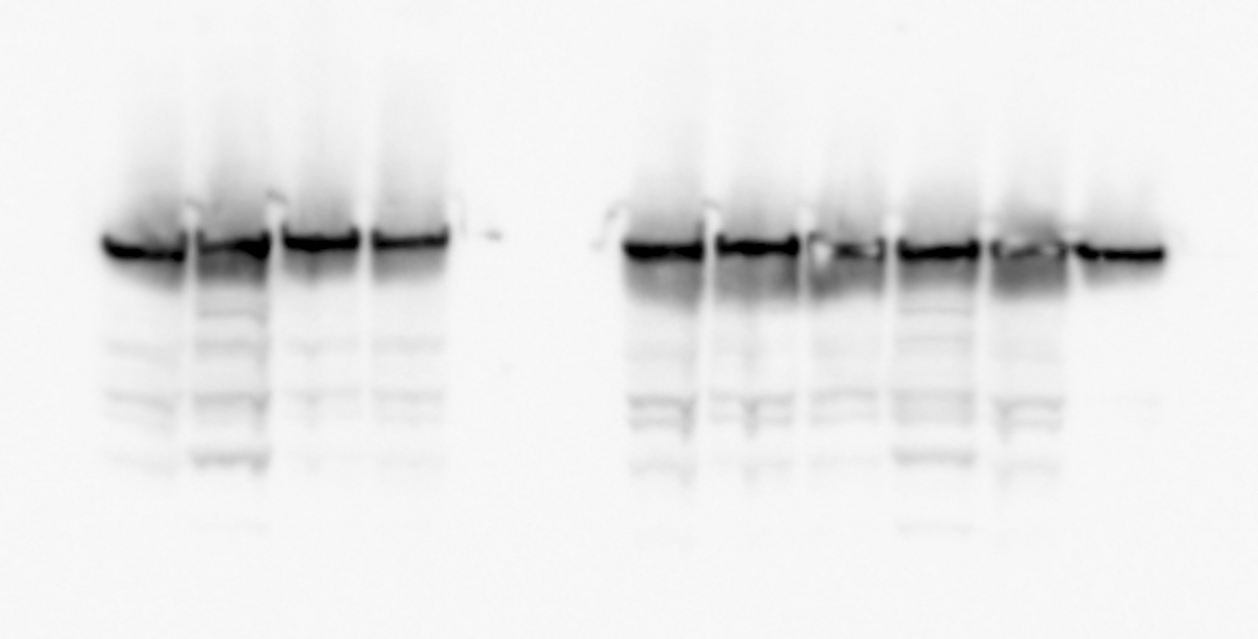


## Notes

Present address: Diego Pasini, European Institute of Oncology, IFOM-IEO Campus, Via Adamello 16, 20139 Milan, Italy.
